# Influence of EGCG (*Epigallocatechin Gallate*) on Physicochemical–Rheological Properties of Surimi Gel and Mechanism Based on Molecular Docking

**DOI:** 10.3390/foods13152412

**Published:** 2024-07-30

**Authors:** Fengchao Zhou, Wenting Jiang, Han Tian, Liuyun Wang, Jiasi Zhu, Wei Luo, Jie Liang, Leiwen Xiang, Xixi Cai, Shaoyun Wang, Qiming Wu, Honglai Lin

**Affiliations:** 1 Fujian Province-Indonesia Marine Food Joint Research and Development Center, Fujian Polytechnic Normal Univeristy, Fuzhou 350300, China; zhoufengchao2005@ptu.edu.cn (F.Z.); wendyjiang316@163.com (W.J.); 220410026@fzu.edu.cn (W.L.); xiangleiwen@163.com (L.X.); 2 Fujian Provincial Key Laboratory of Ecology-Toxicological Effects & Control for Emerging Contaminants, College of Environmental and Biological Engineering, Putian University, Putian 351100, China; 15060388545@163.com (L.W.); 17759988819@163.com (J.Z.); ptxylj0321@126.com (J.L.); 3 Institute of Food and Marine Bio-Resources, College of Biological Science and Engineering, Fuzhou University, Fuzhou 350108, China; tianhan1121@163.com (H.T.); shywang@fzu.edu.cn (S.W.); 4 Fujian Province Yaming Food Co., Ltd., Putian 351100, China; 1648913321@163.com (Q.W.); 22839864@163.com (H.L.)

**Keywords:** surimi, EGCG, gel properties, rheological properties, molecular docking

## Abstract

The influence of epigallocatechin gallate (EGCG) on the physicochemical–rheological properties of silver carp surimi gel was investigated. The gel strength, texture, water-holding capacity (WHC), dynamic distribution of water, and rheological properties of surimi gels added with different levels (0, 0.02, 0.04, 0.06, 0.08, and 0.1%) of EGCG were measured. The results showed that with the increase of EGCG content, the gel strength, hardness, WHC, and immobilized water contents of surimi gels showed a trend of first increasing and then decreasing, and EGCG 0.02% and EGCG 0.04% showed better gel performance as compared with the control. EGCG 0.02% had the highest gel strength (406.62 g·cm), hardness (356.67 g), WHC (64.37%), and immobilized water contents (98.958%). The gel performance decreased significantly when the amounts of EGCG were higher than 0.06%. The viscosity, G′, and G″ of the rheological properties also showed the same trends. The chemical interaction of surimi gels, secondary structure of myofibrillar protein (MP), and molecular docking results of EGCG and silver carp myosin showed that EGCG mainly affected the structure and aggregation behavior of silver carp myosin through non-covalent interactions such as those of hydrogen bonds, hydrophobic interactions, and electrostatic interactions. The microstructures of EGCG 0.02% and EGCG 0.04% were compact and homogeneous, and had better gel formation ability. The lower concentrations of EGCG formed a large number of chemical interactions such as those of disulfide bonds and hydrophobic interactions inside the surimi gels by proper cross-linking with MP, and also increased the ordered β-sheet structure of MP, which facilitated the formation of the compact three-dimensional network gel.

## 1. Introduction

Surimi is a deep-processing, aquatic raw material product with high protein and low fat, and it is a stable fish myofibrillar protein concentrate [1,2]. Gel characteristics are the key indicators to evaluate the quality and consumer acceptance of surimi products. Silver carp is widely cultured in China because it is a freshwater fish that has the characteristics of rapid growth, high yield, and low price, and can be used as a raw material for surimi [3]. However, the myosin content of freshwater fish that form surimi gel is low and prone to gel degradation, thus limiting the development of freshwater fish surimi products processing industry [4]. How to improve the gel characteristics of freshwater surimi through scientific means is an important scientific problem in modern aquatic product processing.

The addition of polyphenols to the surimi can significantly improve the breaking force of the surimi gel [5], and the polyphenols also can form covalent and non-covalent interactions with MP, enhance protein cross-linking, cause changes in gel performance, and are also widely used in the application research of surimi products. Arsyad et al. [6] added olive leaf powder containing phenolic substances to red snapper fish surimi, which enhanced the cross-link of MP, and significantly increased the fracture stress of the surimi gel. However, some studies have also found that polyphenols interacted with MP to form complexes, which leads to the poor texture and water retention characteristics of MP gel and meat products, and has adverse effects on the structure, function, and nutritional characteristics of the proteins. Jongberg et al. [7] added green tea extract to minced pork meat, which increased the cooking loss rate of minced meat products. Jia et al. [8] showed that the gel strength of MP modified with catechin was reduced. Therefore, the use of polyphenols as effective coagulants and natural antioxidants in minced meat products and even meat products also needs to solve the problem of interaction between polyphenols and proteins in food.

EGCG is derived from the catechin monomer in green tea. It is also the main component of tea polyphenols and superior to other catechins in nature and function [9]. The addition of EGCG could effectively inhibit the degradation of texture performance and gel strength of silver carp surimi gel [10]. The molecular structure of EGCG contains a large number of hydroxyl groups, which can form a cage structure complex with MP to bind a large number of water molecules, while reducing the influence of water-soluble proteins on the surimi gel, thus improving the gel strength and making the gel structure more compact [11]. The interaction of polyphenols and MP have significant effects on the secondary and tertiary structure of MP, and these effects vary according to the concentration and chemical structure of polyphenols [12]. Therefore, the interaction mechanism between EGCG and MP in surimi should be clarified.

The influence of EGCG on the physicochemical–rheological properties of silver carp surimi gel were investigated. The 3D structure of silver carp myosin heavy chain (SCMHC) was constructed through homology modeling, and the binding ability and sites of EGCG and SCMHC were used to explore by molecular docking technology, and revealed the intrinsic connection and molecular mechanism. The aim is to provide a theoretical basis for the development of new surimi products and gel quality control technology.

## 2. Materials and Methods

### 2.1. Materials

Frozen silver carp surimi was provided by Fujian Province Yaming Food Co., Ltd. (Putian, China). EGCG was purchased from Shanghai Yuanye Bio Technology Co., Ltd. (Shanghai, China) and its purity was 98%. All other chemicals were of analytical grade.

### 2.2. Preparation of Surimi Gel

Five portions of thawed surimi were taken and 200 g of each portion was stirred in a cutmixer (MJ-LZ25, Guangdong Midea Household Electrical Appliance Manufacturing Co., Ltd., Guangzhou, China) for 2 min, before NaCl with 2% (*w*/*w*) was added and stirred for 3 min. Then, EGCG with quality fractions of 0.02%, 0.04%, 0.06%, 0.08%, and 0.1% was added and stirred for 3 min. The stirring process controlled the temperature of surimi and we kept it below 10 °C by adding ice water (the quality of ice water was 80% of the mass of surimi).The surimi sols obtained after the end of stirring were equilibrated at 25 °C for 30 min. Accurately weighed 10 g samples of surimi sol were placed into the weighing bottle (30 mm × 50 mm), and the heat-induced gel was heated in a two-stage water bath (40 °C/30 min, 90 °C/20 min), then cooled by ice water to room temperature. The final obtained surimi gel samples were characterized as EGCG 0.02%, EGCG 0.04%, EGCG 0.06%, EGCG 0.08%, and EGCG 0.1%, respectively, and the control surimi gel sample had no added EGCG.

### 2.3. Determination of Surimi Gel Texture

Texture profile analysis (TPA) and gel strength of the surimi gels were measured using a texture analyzer (CT3, Brookfield Corporation, New York, NY, USA) equipped with probes TA5 and TA18, respectively. The TPA test parameters were pre-test speed 3 mm/s, test speed 2 mm/s, post-test speed 3 mm/s, compression strain 30%, and trigger force 5 g, respectively. The test indicators of TPA were hardness, cohesiveness, springiness, and chewiness. The gel strength test parameters were distance of press down 20 mm, pre-test speed 3 mm/s, test speed 2 mm/s, post-test speed 3 mm/s, respectively. The breaking force (g) and deformation (mm) were measured, and the gel strength value (g·mm) was the product of breaking force and deformation.

### 2.4. Determination of WHC of Surimi Gels

WHC was measured using the method described by Liang et al. [13] with some modification. Surimi gels were sliced into cylindrical samples with 5 mm diameter and weighed (m_1_). Then, they were wrapped in two layers of filter paper and transferred to a 10 mL centrifuge tube, and the samples were centrifuged at 8000 r/min for 10 min. Finally, the samples were weighed again (m_2_) after the removal of filter paper. WHC was calculated using Formula (1):

WHC (%) = (m_2_/m_1_) × 100 (1)

### 2.5. Rheological Measurement

Static rheological properties: the viscosity and thixotropy of surimi sol samples were measured at 20 °C with a rheometer (ViscotesterIQ, Thermo Fisher Scientific, Waltham, MA, USA) equipped with a rotor (FL224B/SS-01160184). Test parameters are shown in Table 1.

Dynamic rheological properties: 2–3 g of surimi sol was placed on a rotating rheometer (MCR302, Anton Paar, Graz, Austria) with a parallel plate (PP25), and tested by temperature scanning using sinusoidal oscillation patterns. The test parameters were 25 °C to 90 °C, heating rate 5 °C/min, shear strain 1%, angular frequency 10 rad/s, respectively. The plate slit spacing was 1 mm, and the slit was sealed with silicone oil to prevent sample steaming. The storage modulus (G′), loss modulus (G″) and tanδ (G″/G′) were recorded versus temperature.

### 2.6. Low-Field Nuclear Magnetic Resonance (LF-NMR)

The lateral relaxation time T_2_ was detected using an LF-NMR analyzer (Minispec MQ20, Bruker, Ettlingen, Germany) with the surimi gel placed at 20 mm in height into a 10 mm diameter NMR tube and subsequently inserted into the analyzer and operated at 20 MHz.

### 2.7. Determination of Chemical Interaction

As noted, the method of Yan et al. [14] was used with some modification. Briefly, 1 g surimi gel was added to 10 mL 0.05 mol/L NaCl solution (A) and homogenized for 2 min. The aforesaid homogenate was stirred at 4 °C in an ice water bath for 1 h, then the mixture was centrifuged for 15 min at 10,000 g, and the supernatant was collected (SA). The precipitate was added to 10 mL 0.6 mol/L NaCl solution (B), and the procedure was repeated. Then, 10 mL 0.6 mol/L NaCl + 1.5 mol/L urea (C), 0.6 mol/L NaCl + 8 mol/L urea (D) and 0.6 mol/L NaCl + 8 mol/L urea + 0.5 mol/L β-mercaptoethanol (E) were successively added to the precipitate. Supernatants (SB, SC, SD, and SE) were collected after homogenization, stirring, and centrifugation. The protein concentration of the supernatant was determined using the biuret method. Ionic bonds, hydrogen bonds, hydrophobic interactions, and disulfide bonds were represented by the protein concentration in the supernatant of SB-SA, SC-SB, SD-SC, and SE-SD, respectively.

### 2.8. Fourier Transform Infrared Spectroscopy (FT-IR)

The secondary structures of MP in surimi gels were analyzed using FT-IR (TENOSOR27, Bruker, Germany). The surimi gels were freeze-dried and ground into powder. An amount of 1.5 mg of powder was mixed with 150 mg KBr and then pressed into a sheet. Samples were scanned in the wave number range of 400–4000 cm^−1^ with resolution of 4 cm^−1^. Fourier self-deconvolution and spectral line fitting techniques were used to handle the changes of the inferred protein secondary structure, and the quantitative calculation of the protein secondary structures was performed with PeakFit software Version 4.

### 2.9. Scanning Electron Microscopy (SEM)

Surimi gels were cut into small cubes (side: 3 mm × 5 mm × 3 mm). The cubes were fixed for 24 h with 2.5% glutaraldehyde, then dehydrated in ethanol at serial concentrations of 30%, 50%, 60%, 70%, 80%, 90%, and 100% (*v/v*). Subsequently, the prepared samples were freeze-dried in a vacuum (SCIENTZ-10N, Ningbo Xinzhi Biotechnology Co., Ltd., Ningbo, China), sputter-coated with gold and then observed using a scanning electron microscope (SU8010, Hitachi Co., Tokyo, Japan).

### 2.10. Homology Modeling of the SCMHC

Silver carp myosin sequences were obtained from the NCBI (https://www.ncbi.nlm.nih.gov/ (accessed on 1 August 2023)). Homology modeling was performed using the SWISS-MODEL (https://swissmodel.expasy.org/ (accessed on 1 August 2023)), and the highest scoring result was selected. Subsequently, the modeling results were evaluated using the Ramachandran plot, VERIFY 3D, and ERRAT in SAVESv6.0 (https://saves.mbi.ucla.edu/ (accessed on 1 September 2023)).

### 2.11. Molecular Docking of EGCG and SCMHC

The molecular structure of the ligand EGCG was obtained from the pubchem website (https://pubchem.ncbi.nlm.nih.gov/ (accessed on 1 August 2023)). The molecular docking of EGCG and SCMHC were performed by Discovery Studio 2019, and the docking result with the highest score was selected for further analysis.

### 2.12. Statistical Analysis

For each batch of samples, all specific experiments were carried out in triplicate. All data were analyzed using the general linear models procedure in the Statistix 8 version 8.1 software package (Analytical Software, St. Paul, MN, USA). Analysis of variance was performed to determine the significance of the main results. Graphs were generated by Original 8.5.

## 3. Results

### 3.1. TPA and Gel Strength of Surimi Gels

TPA and gel strength tests are effective methods to study the texture properties of gels and have been widely used in the study of physical characteristics of surimi gels [15]. Effects of different amounts of EGCG on texture properties of surimi gels are shown in Table 2. The surimi gels to which 0.02% and 0.04% EGCG were added had significantly increased gel strength and hardness compared with the control, and the gel strength and hardness of EGCG 0.02% were highest. However, the gel strength and hardness showed a significant decreasing trend when EGCG amounts of more than 0.06% were added, and EGCG 0.08% and EGCG 0.1% had significantly lower values as compared to the control. Cohesiveness, springiness, and chewiness had the same change trends with the increase of EGCG amounts. Gel strength and hardness are important parameters for evaluating the quality of surimi gel products, which reflect the ability of surimi to form heat-induced gels [16]. It follows that lower concentrations of EGCG helped to promoted surimi gel formation, whereas higher concentrations of EGCG were unfavorable for the gel formation. The lower concentrations of polyphenols were able to enhance the hydrophobic interactions and facilitate the gelation of surimi proteins [17]. However, the high concentration of polyphenols caused hydrophobic aggregation of proteins, hindered the cross-link between proteins, and made surimi protein form a disordered and loose network structure, thus reducing the texture characteristics of surimi gel [18].

### 3.2. Water-Holding Capacity (WHC) of Surimi Gels

**Figure 1:**
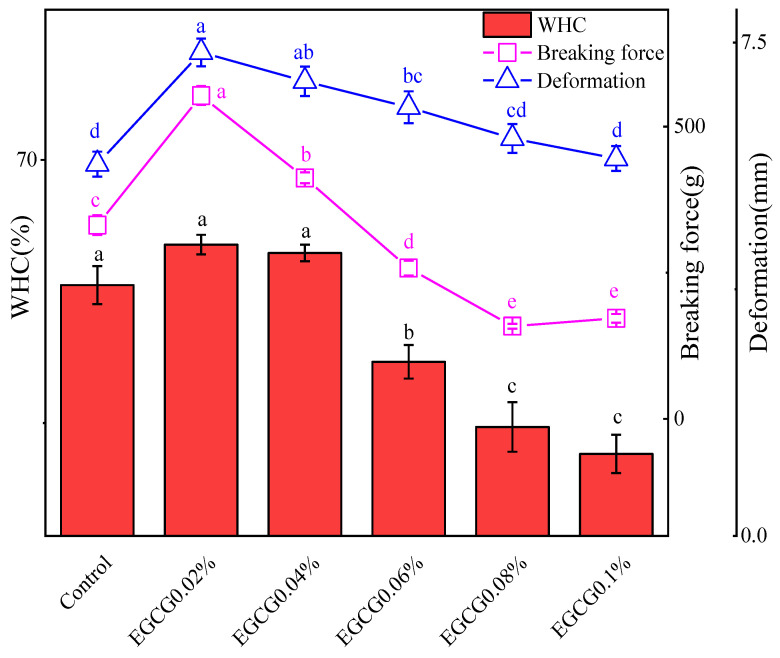
WHC, breaking force, and deformation of surimi gels with different amounts of EGCG (the same indicator annotated with different letters indicates the significant difference (*p* < 0.05); the same letters indicate the difference is not significant (*p* > 0.05)).

Effects of different amounts of EGCG on WHC of surimi gels are shown in Figure 1, and breaking force and deformation of the gels are also plotted in the figure. WHC is the ability of the MP in surimi gel to retain water after being heated to form a three-dimensional network gel structure. Generally, the denser the gel network structure, the better its ability to retain water, and the higher the WHC. The breaking force represents the closeness of the internal structure of the gel, and the deformation represents the flexibility and elasticity of the gel [19], so they were all correlated with the WHC. As can be seen from Figure 1, the three indicators had the same change trends. EGCG 0.02% and EGCG 0.04% showed significantly higher WHC, breaking force, and deformation than the control, and the EGCG 0.02% had the highest of the three indicators. With the amounts of EGCG higher than 0.06%, all the three indicators showed significant downward trends, and the EGCG0.1% had lowest of the three indicators. The WHC of EGCG 0.1% (50.45%) decreased by 11.23% compared to the control (61.68%). These results suggest that lower concentrations of EGCG facilitated the surimi gel formation, whereas the higher concentration of EGCG caused disruption of the gel. The lower concentration of polyphenols facilitated the formation of the gel because the polyphenols formed non-disulfide bond covalent cross-links with sulfydryl, and polyphenols acted as connections between proteins, thereby increasing the aggregation of protein molecules [18,20]. The higher concentrations of polyphenols were bound to protein molecules excessively, destroyed the protein spatial structure and caused poor aggregation, leading to a loose gel network and decreasing the WHC of the system [17].

### 3.3. Rheological Properties

#### 3.3.1. Static Rheological Properties

**Figure 2:**
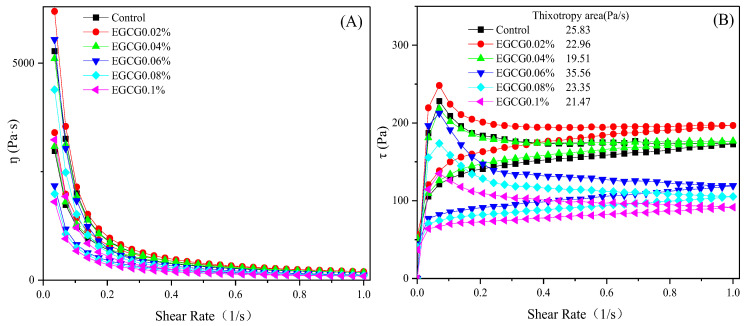
Static rheological curve ((**A**): viscosity, (**B**): thixotropy) of surimi sols with different amounts of EGCG.

Figure 2A shows the relationship of the viscosity and shear rate of surimi sols with different amounts of EGCG. The viscosity of all samples decreased rapidly with the increasing of the shear rate; this indicated that surimi sols had shear thinning behavior and showed pseudoplasticity. During the shear process, the surimi sol showed deformations include orientation, extension, dispersion, etc., which reduced the flow resistance and caused the shear thinning [21]. Viscosity is a property blocking the flow or deformation of surimi sol, and resulted from the cohesion and molecular diffusion between protein and protein and between protein and water in surimi sols. The interaction between EGCG and MP molecules also affected the viscosity of the surimi sols. With the increase of EGCG added, the viscosity of surimi sols increased first and then decreased, and the viscosity of EGCG 0.02% was the largest and higher than that of the control. When the amount of EGCG was greater than 0.06%, the viscosity of the surimi sols was decreased and lower than that of the control, and the viscosity of EGCG 0.1% was minimal. The trend of the viscosity of surimi sols was the same as that of the gel strength and the hardness.

Figure 2B shows the thixotropy of surimi sols denoted by changes of shear stress. Thixotropy is one of the most important rheological properties of sol and closely related to the quality of food. Under the external shear force, the internal network structure of surimi sol will be damaged. Due to the shearing, there were differences between the rate of the destruction of the sol structure and the rate of recovery of the sol itself, and a clockwise loop called thixotropic rings formed, which consisted of the upline and downline when the shear stress changed with the shear rate [22]. The thixotropic curves of the control and surimi sols with EGCG were all clockwise thixotropic rings, and indicated that all surimi sols belonged to the thixotropic system. The size of the thixotropic ring area indicates the shear stability of the system, and the smaller the area, the better the stability of the system [23]. Good stability of the surimi sol system contributes to the formation of surimi gel.

The thixotropic ring areas of EGCG 0.02%, EGCG 0.04%, EGCG 0.08%, and EGCG 0.1% were significantly reduced compared with the control. The shear stresses corresponding to each point on the thixotropic ring curve of EGCG 0.02% were higher than those of the control, while the shear stresses of EGCG 0.04% were close to those of the control, and the shear stresses of EGCG 0.06%, EGCG 0.08%, and EGCG 0.1% showed a significant downward trend with the increase of the EGCG amount. The reason for these changes may be due to the interaction of EGCG with MP molecules in surimi sol. The lower concentrations of EGCG (0.02–0.04%) were able to enhance the interaction of MP–MP molecules in the sol network to increase or maintain the shear stress, whereas the higher concentration of EGCG (0.06–0.1%) disrupted this interaction to reduce the shear stress. Both the increase of the shear stress and the decrease of the thixotropic ring area contributed to promoting the surimi gel formation. This was also the reason why the gel strength and hardness of EGCG 0.02% were higher than those of the other samples.

#### 3.3.2. Dynamic Rheological Properties

The G′, G″, and tanδ of surimi sols reflected the changes in their viscoelasticity during heating to form a gel [24], and also reflected the strength or weakness of gel properties in different stages of the MP structure unfolding and aggregation in surimi. G′ and G″ represent the elasticity and viscosity of the samples, respectively. The higher the G′, the better the gelation ability of MP in the sample [25]. Tanδ = G″/G′; when the tanδ value was greater than 1, the sample viscosity was greater than the elasticity, which appeared as the viscosity fluid; when the Tanδ value was less than 1, the sample elasticity was greater than the viscosity, which appeared as sol or gel.

**Figure 3:**
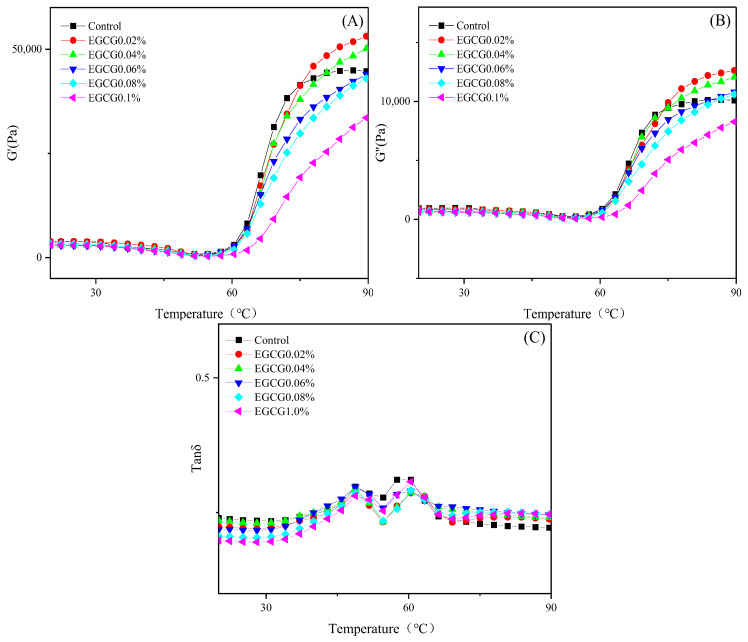
Dynamic rheological curve ((**A**): G′; (**B**): G″; (**C**): tan δ) of surimi sols with different amounts of EGCG.

Figure 3A shows that the G′ of all samples at the start of the heating phase decreased with increasing temperature, and reached a minimum at 50–57 °C. The gel degradation and collapse of MP in surimi occurred at about 50 °C, and the light chain of myosin dissociated, which enhanced the molecular fluidity, so the G′ value decreased [26,27]. At 57–90 °C, the myosin heavy chain and actomyosin underwent heat denaturation to form an irreversible gel network structure, and the G′ value began to rise [28]. Figure 3B shows that the trends of G″ were similar to those of G′ but at much lower values than G′, due to the higher elastic component in the gel system formed by surimi sol during heating. Figure 3C shows that all samples had tanδ values less than 1, and indicates that a mainly elastic surimi gel was formed during heating.

In the high temperature stage of the gel formation (80–90 °C), the G′ values of EGCG 0.02% and EGCG 0.04% were higher than those of the control, and that of the EGCG 0.2% was the highest. However, EGCG 0.06%, EGCG 0.08%, and EGCG 0.1% showed lower G′ values than the control, and that of the EGCG 0.1% was the lowest. These indicated that the gelation ability of surimi sols changed from strong to weak with increase of the EGCG amount. The gelation ability of surimi sol was determined by MP, while the stability of MP was influenced by myosin. The weakening of myosin head interactions was the main reason for the decrease in MP stability [18], while the decrease in the stability directly affected the heat-induced gelation process of MP adversely.

The increase of G′ of the surimi sols with lower concentration of EGCG added may be due to the EGCG promoting the unfolding of myosin molecules and enhancing the cross-linking between protein and protein, and between protein and EGCG, resulting in a more stable heat-induced gel structure being formed. The G′ of the surimi sols reduced significantly when the amounts of EGCG were higher than 0.06%, which indicated the protein cross-linking action of the three-dimensional network gel was weakened. This was mainly due to the fact that the higher concentration of EGCG caused denaturation and aggregation of MP through the generation of a sulfhydryl-quinone adduct and promoted the exposure of hydrophobic groups, hindering the MP in forming a three-dimensional network gel structure, thus reducing the G′ [29]. Jiao et al. [30] proposed that the moderate concentration of EGCG had an improvement effect on MP gel, which may be due to the cross-linking of the phenolic hydroxyl group of EGCG with amino acid residues such as lysyl and cysteinyl of myosin molecules.

### 3.4. Moisture Mobility of Surimi Gels

**Figure 4:**
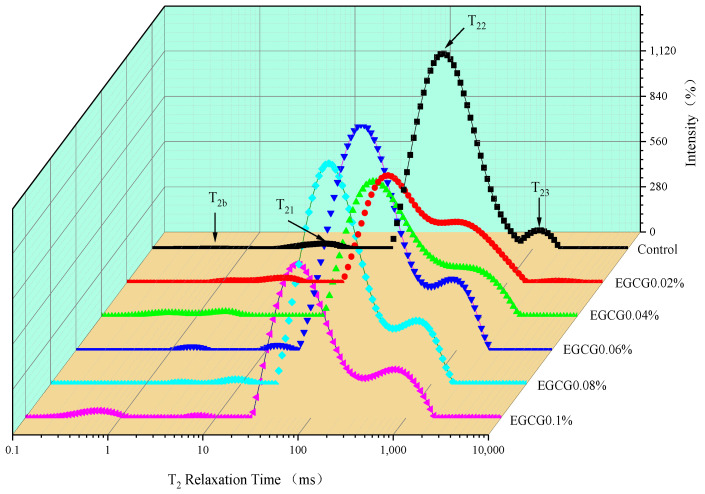
Effects of EGCG addition on the T_2_ relaxation time of surimi gels.

The LF-NMR T_2_ relaxation time technique was used to analyze the dynamic distribution of water inside the gel effectively [31]. Figure 4 shows the T_2_ relaxation time profiles fitted by the LF-NMR attenuation curve of the control and surimi gels with different concentrations of EGCG. Four relaxation peaks appeared at the T_2_ relaxation time profiles for all samples, representing the water distribution and migration of the different components in the gels, at positions of about 0.1–1 ms (T_2b_), 1–10 ms (T_21_), 100–1000 ms (T_22_), and 1000–10,000 ms (T_23_), respectively [2]. These four components were classified to three water states in the gels; namely, bound water(T_2b_), immobilized water (T_21_ and T_22_), and free water (T_23_), respectively. The bound water was tightly bound to non-aqueous substances in the gel system; the immobilized water represented by T_21_ had weak mobility and was inside the gel network structure, while the immobilized water represented by T_22_ had certain mobility and was between the gel network structures; and the free water was more mobile and easily removed from the gel system [32]. The largest T_22_ peak appeared in all samples in Figure 4 represented immobilized water. However, the T_23_ relaxation peaks of EGCG 0.02% and EGCG 0.04% were shifted to the fast relaxation direction on the left and merged with the respective T_22_ relaxation peaks. These indicated that after the addition of 0.02% and 0.04% EGCG, the free water in the surimi gels was converted to immobilized water. The immobilized water accounted for the vast majority of the water in the surimi gel, which depended on the change in the three-dimensional network gel structure, an important indicator that directly determined the WHC.

The starting times of T_2_ relaxation peaks and the peak area proportions of the three components water for each gel samples are listed in Table 3 (P_2b_ represents bound water, P_21_ and P_22_ represent immobilized water, and P_23_ represents free water). The T_2_ relaxation time indicates the strength of the interaction between MP and the surrounding water molecules in surimi gel, and the shorter relaxation time indicated faster mutual binding and thus a stronger interaction [33]. The longer the relaxation time, the less the binding force of water molecules with the gel; that is, the worse the water stability and easier the flow. There were no significant differences (*p* > 0.05) between the starting time of the T_2b_ relaxation peak in surimi gels with different amounts of EGCG and the control. However, the starting time of the T_22_ relaxation peak for surimi gels with EGCG was significantly reduced (*p* < 0.05) as compared with the control. Compared with the control, the P_2b_ of surimi gels showed a significantly higher trend (0.556–2.309%) with the increase of EGCG amount. These changes may be due to the EGCG molecules containing a great quantity of hydroxyl groups that combine with water molecules [2], thus increasing the proportion of bound water in the gel, while also shortening the T_22_ relaxation time. P_21_ and P_22_, which represent immobilized water, showed different changes after the EGCG was added to the surimi gel. The immobilized water in EGCG 0.02% (98.958%) and EGCG 0.04% (97.781%) increased significantly (*p* < 0.05) compared to the control, and then decreased significantly (83.768–78.396%) with the increase of EGCG amount (0.6–0.1%). The P_23_ trend was opposite to the change in P_21_ and P_22_; the free water in EGCG 0.02% (0.486%) and EGCG 0.04% (1.126%) decreased significantly (*p* < 0.05) compared to the control, and then increased significantly (14.997–19.295%) with the increase of EGCG amount (0.06–0.1%). The lower concentrations of EGCG were able to promote the formation of the surimi gel and further convert the free water into immobilized water, and maintained more water in the three-dimensional network gel structure. However, the gel system formed by the interaction of MP with EGCG at a higher concentration led to a reduction in the contact frequency between protein and protein molecules, which broke the protein gel network structure and reduced the ability to maintain water [34,35]. This was also the reason for the significant increase in the free water of surimi gels when the EGCG amount was greater than 0.06%.

### 3.5. Determination of Chemical Interaction

**Figure 5:**
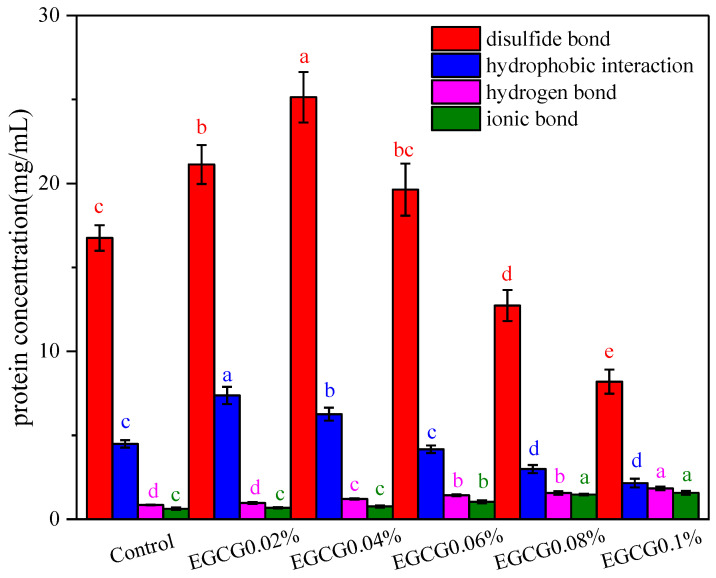
Effects of EGCG addition on chemical interactions of surimi gels (the same indicator annotated with different letters indicate a significant difference (*p* < 0.05); the same letters indicate the difference is not significant (*p* > 0.05)).

The chemical interactions that affected the structural stability of the surimi gel included those of disulfide bonds, hydrophobic interactions, hydrogen bonds, and ionic bonds, etc. Figure 5 shows the effect of different amounts of EGCG on the internal chemical interactions of the surimi gel. It was obvious that the disulfide bond was the main chemical interaction in the surimi gel, followed by the hydrophobic interaction, hydrogen bonds, and ionic bonds, represented a small proportion of the chemical interactions. Disulfide bonds were formed by the oxidation of sulfhydryl groups of two cysteine residues within or between the peptide chains. The disulfide bond contents of EGCG 0.02% and EGCG 0.04% were significantly higher compared with the control, and EGCG 0.04% had the highest (*p* < 0.05), but after the EGCG amounts added were higher than 0.06%, the disulfide bond contents in the surimi gel decreased significantly, and that in EGCG0.1% was the lowest (*p* < 0.05). This may be due to the low added amounts of EGCG (0.02% and 0.04%) connecting the MP molecules and the non-disulfide covalent cross-linking with the sulfhydryl group, which unfolded the MP molecules moderately, and oxidized the sulfhydryl group in the peptide chain to form the disulfide bond; then, the polymerization of the MP and the disulfide bond contents were increased, which facilitated the formation of the surimi gel [18,20]. The high added amounts of EGCG would over-cross-link with MP molecules, which could affected the oxidation of sulfhydryl groups to form disulfide bonds and was unfavorable to surimi gel [36]. Hydrophobic interactions were the main driving force in protein folding, and affected the structure and characteristics of most protein molecules [37]. The hydrophobic interactions of the surimi gels were increased first and then decreased with the increase of EGCG amount; those of the EGCG 0.02% and EGCG 0.04% were significantly higher compared with the control, and EGCG 0.02% showed the highest (*p* < 0.05), while when the EGCG amounts were higher than 0.06%, the hydrophobic interactions in the surimi gel were decreased significantly, and EGCG 0.1% had the lowest (*p* < 0.05). This was also due to the lower concentration of EGCG promoting proper unfolding of the protein, while the higher concentrations of EGCG were over-cross-linked with MP, and thus caused aggregation and denaturation of the MP molecules, and further folded to bury the hydrophobic amino acids in the interior.

### 3.6. FT-IR Analysis

**Figure 6:**
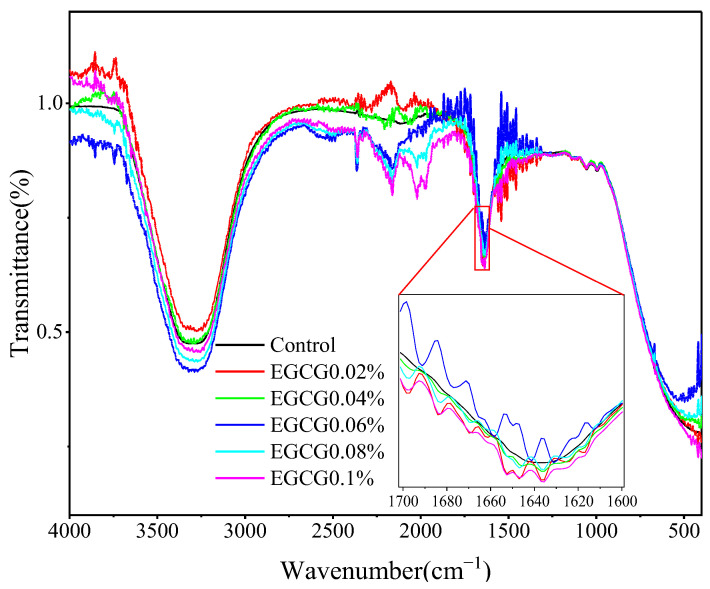
FT-IR of surimi gels with different amounts of EGCG.

To further investigate whether EGCG affected the secondary structure of MP in the surimi gels, they were analyzed by FT-IR, as shown in Figure 6. Most of the protein secondary structure changes were analyzed by spectral changes of FT-IR in amide-I band (1700–1600 cm^−1^) [38]. The characteristic frequency of the amide I band can be used to represent the secondary structure of proteins, mainly including C=O stretching vibration and C-N stretching vibration [39]. The absorption peak corresponds to the protein secondary structure as follows: 1610–1640 cm^−1^ corresponds to a β-sheet, 1640–1650 cm^−1^ corresponds to a random coil, 1650–1658 cm^−1^ corresponds to a α-helix, and 1660–1695 cm^−1^ corresponds to a β-turn [40].

**Figure 7:**
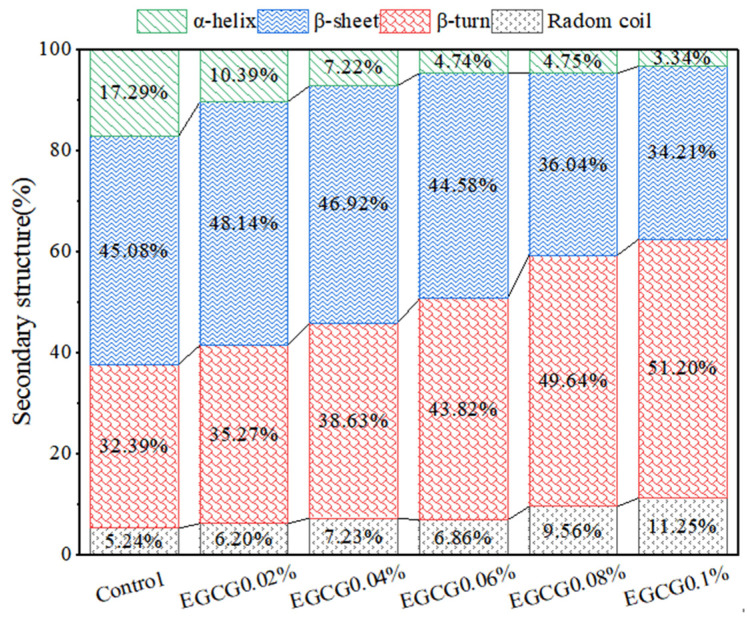
Changes in MP secondary structures of surimi gels with different amounts of EGCG.

The effects of EGCG on the secondary structure of MP in surimi gels were analyzed by FT-IR, as shown in Figure 7. The proportions of MP secondary structures in the control group were α-helix 17.29%, β-sheet 45.08%, β-turn 32.39%, and random coil 5.24%, respectively. After the EGCG was added, the proportion of α-helices decreased with increase of EGCG content (10.39–3.34%); the proportion of β-sheet first increased and then decreased, in which EGCG 0.02% (48.14%) and EGCG 0.04% (46.92%) were higher than the control. EGCG 0.02% was the highest, and then continued to decrease with the increase of EGCG content (44.58–34.21%); the proportion of β-turn and random coil were increased with increase of EGCG content. These results showed that α-helix in the MP secondary structure had a tendency to convert into β-sheet, β-turn, and random coil when EGCG amounts were lower (0.02% and 0.04%), and α-helix and β-sheet had a tendency to convert into β-turn and random coil when EGCG amounts were higher (0.06–0.1%). The conversion of the α-helix to an ordered β-sheet structure was more favorable to form a regular gel network, and thus improved the texture and water retention of the gel [41]. This was also why the lower concentrations (0.02% and 0.04%) of EGCG significantly increased the gel strength, hardness, and WHC of the surimi gels. It has also been suggested that the conversion of α-helix to β-turn and random coil are responsible for the decrease in gel texture and water retention [41]. The increase of random coil structure made a disordered aggregation of MP molecules, which reduced the degree of cross-linking between MP molecules, resulting in uneven, rough, and large mesh structures, and reduced the gel strength [42]. The increased of β-turn and random coil at higher EGCG concentrations also resulted in the disordered aggregation of MP molecules, which accounted for the significant decreased in gel strength, hardness, and WHC of the surimi gels.

### 3.7. Microstructure

**Figure 8:**
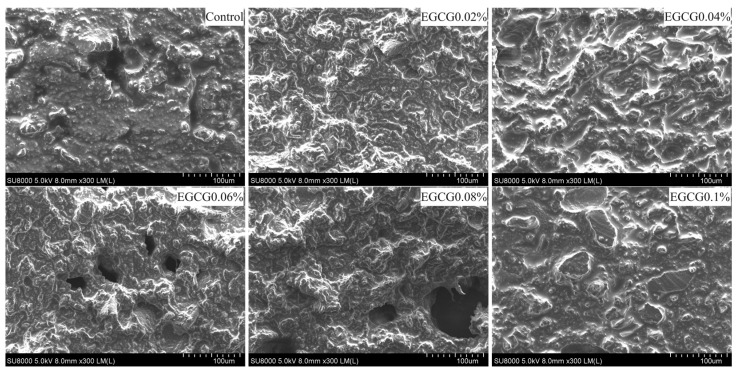
Scanning electron microscopic photographof surimi gels with different amounts of EGCG.

Microstructure is an intuitive reflection of the surimi gel state, are described in Figure 8. The control had uneven structures, large pores, and irregular shape. The microstructures of EGCG 0.02% and EGCG 0.04% were compact and homogeneous, and had better gel formation ability. However, when the amount of EGCG increased to 0.06–0.1%, the surimi gel network structure changed significantly: the gel surface gradually became rough, loose, and uneven, the pores became larger and larger, and the gel structure of EGCG 0.1% was broken and accompanied by the lamellar structure. This was mainly due to the stronger interaction between higher concentration of EGCG and MP, which hindered the cross-linking between protein molecules, and the protein network structure was poor, and ultimately could not form a better gel network structure. These result were consistent with the fact that the higher concentration of EGCG worsened the gel strength, WHC, and rheological properties.

### 3.8. Evaluation of Modeling Results

**Figure 9:**
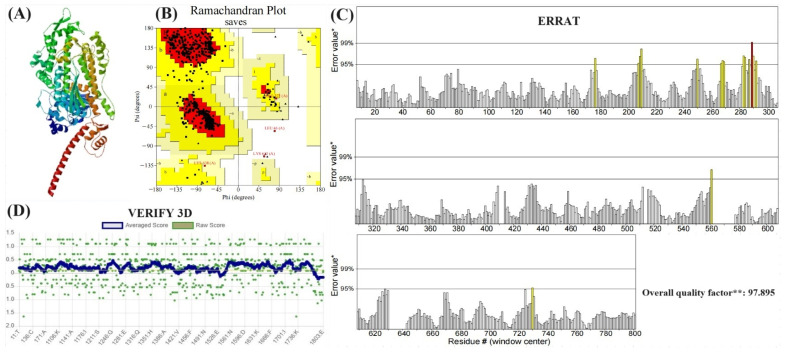
Evaluation of SCMHC modeling results. (**A**) SCMHC results; (**B**) Ramachandran plot; (**C**) ERRAT (* On the error axis, two lines are drawn to indicate the confidence with which it is possible to reject regions that exceed that error value; ** Expressed as the percentage of the protein for which the calculated error value falls below the 95% rejection limit. Good high resolution structures generally produce values around 95% or higher. For lower resolutions (2.5 to 3A) the average overall quality factor is around 91%.); (**D**) VERIFY 3D.

Homology modeling of SCMHC constructed by SWISS-MODEL and the 3D structure model are shown in Figure 9A. The modeling results were assessed by SAVESv6.0 (https://saves.mbi.ucla.edu/ (accessed on 1 September 2023)) in order to further illustrate the plausibility. Ramachandran plot is a visualization method to describe whether the dihedral angles (ψ and φ) of amino acid residues are in reasonable regions in the protein structure, and can also reflect whether the conformation of the protein is reasonable.

As can be seen from the Ramachandran plot in Figure 9B, in the 3D model constructed of SCMHC, 93.0% of amino acid residues fell in the most favoured regions, 6.4% in the additional allowed regions, 0.3% in the generously allowed regions, 0.3% in the disallowed regions, and 99.7% were within reasonable regions. The model of SCMHC passed the VERIFY 3D test. As can be seen in Figure 9D, 89.50% of the residues had averaged scores greater than or equal to 0.2. The accuracy of the three-dimensional structure of protein increased with the increase of the value of the overall quality factor. The overall quality factor of the 3D structure model was 97.895%, which indicated that the homology model of the SCMHC was feasible and could be used in the next molecular docking studies.

### 3.9. Molecular Docking of EGCG and SCMHC

**Figure 10:**
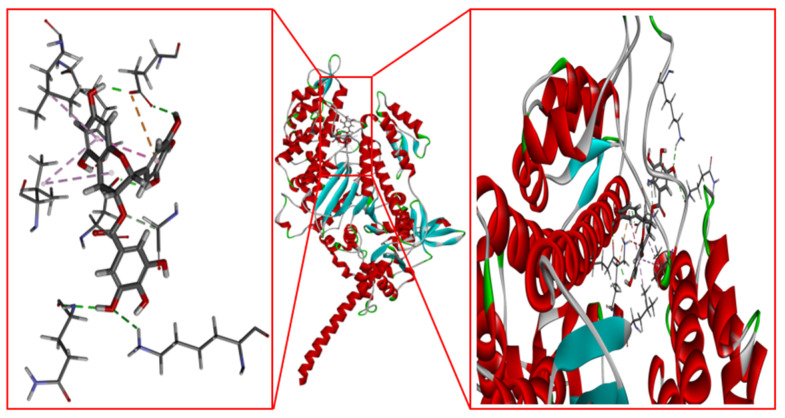
Molecular docking results of EGCG with SCMHC.

The docking results of EGCG and SCMHC are shown in Figure 10. Non-covalent interaction between polyphenols and proteins mainly included those of hydrogen bonds, hydrophobic interactions, van der Waals forces, electrostatic interactions, etc. These forces were generally weaker because they did not involve shared electron pairs and were always reversible [43]. The formation of protein–polyphenol conjugates usually depended on hydrogen bonds and hydrophobic interactions [44].

**Figure 11:**
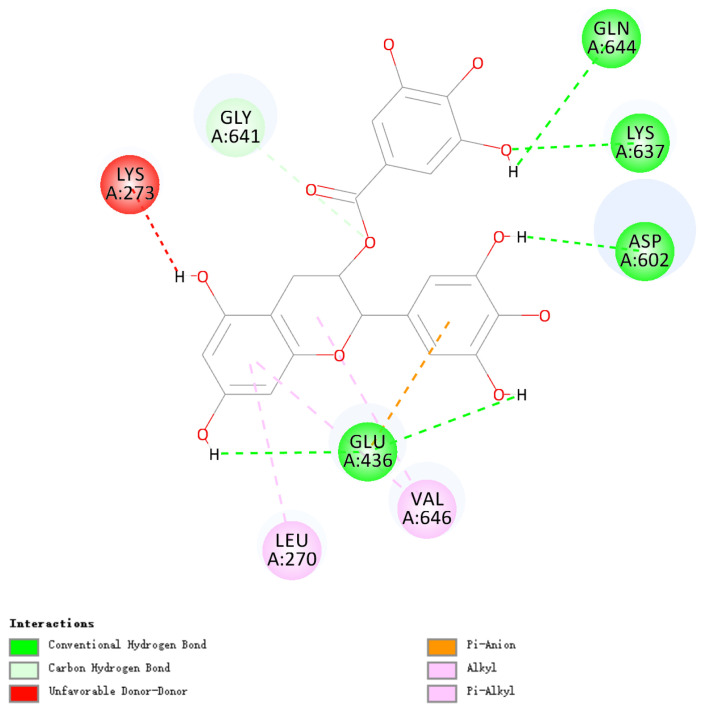
2D diagrams of the molecular docking.

It can be seen from the 2D schematic of the non-covalent interaction (Figure 11) that EGCG interacted with Gln 644, Lys 637, Asp 602, Glu 436, Gly 641, Leu 270, Val 646, etc. Hydrogen bonds were formed between EGCG and Gln 644, Lys 637, Asp 602, Glu 436, and Gly641. Hydrophobic interactions were reflected in the Pi-Alkyls, which occurred between the benzene ring from EGCG and the alkyl group from Leu 270 or Val 646. The Pi-Anion belonging to electrostatic interaction occurred between the benzene ring from EGCG and the anion from Glu 436. These showed that EGCG formed non-covalent interactions with SCMHC mainly through hydrogen bonds, hydrophobic interactions, and electrostatic interactions. The amount of these non-covalent interactions affected the myosin molecular structure changes during MP heat induction, and thus affected the surimi gel properties. The lower concentrations of EGCG formed appropriate amounts of the non-covalent interactions with silver carp myosin molecules, so that the myosin molecules unfolded appropriately and the hydrophobic groups were exposed, and formed more ordered β-sheet structures, which also produced more disulfide bonds and hydrophobic interactions, and were favorable to the surimi gel. However, the higher concentrations of EGCG formed numerous non-covalent interactions with myosin molecules, which had a large impact on the myosin molecular structure, causing further disordered aggregation, folding and denaturation, and also hindered the formation of hydrophobic interactions and disulfide bonds.

## 4. Conclusions

The aim of this study was to explore the intrinsic mechanism of the effect of EGCG on the quality of surimi gel. The physical characteristics of the surimi gels showed that when the EGCG amounts were 0.02% and 0.04%, the gels had higher gel strength, hardness, and water retention, and remained more immobilized water in the three-dimensional network gel structure. The determination of static and dynamic rheological properties showed that the thixotropy and G′ of EGCG 0.02% and EGCG 0.04% were improved significantly. The lower concentrations of EGCG formed a large amount of chemical interactions, such as disulfide bonds and hydrophobic interactions, inside the surimi gel by proper cross-linking with MP, and also increased the ordered β-sheet structure of MP, which facilitated the formation of compact three-dimensional network gel. Meanwhile, the influence mechanism of EGCG on surimi gel quality was further explored by molecular docking technology. Molecular docking results showed that EGCG mainly affected the structure and aggregation behavior of SCMHC through non-covalent interactions such as those of hydrogen bonds, hydrophobic interactions, and electrostatic interactions, and the quality of the surimi gel could be controlled by adjusting the amount of EGCG added. This study is helpful to further understand the mechanism of EGCG promoting or destroying the formation of surimi gel, and it also provide an effective quality control agent for surimi products.

## Figures and Tables

**Table 1:** Test parameters for static rheological properties.

	Rotor Speed (r/s)	Determination Time (s)	Determination of Data Points
Viscosity	0.000001–1	180	30
	1–0.000001	180	30
Thixotropy	0.000001–1	180	30
	1–0.000001	180	30

**Table 2:** Effects of different amounts of EGCG on texture properties of surimi gels.

Gels	Gel Strength (g·cm)	Hardness (g)	Cohesiveness (mJ)	Springiness (mm)	Chewiness (mJ)
Control	187.61 ± 15.94 ^c^	268.67 ± 10.26 ^b^	0.17 ± 0.06 ^bc^	4.82 ± 0.07 ^b^	5.27 ± 0.55 ^b^
EGCG 0.02%	406.62 ± 22.79 ^a^	356.67 ± 10.97 ^a^	0.43 ± 0.05 ^a^	5.19 ± 0.11 ^a^	9.30 ± 0.61 ^a^
EGCG 0.04%	284.81 ± 4.95 ^b^	283.33 ± 9.29 ^b^	0.27 ± 0.05 ^b^	4.57 ± 0.12 ^b^	4.27 ± 0.25 ^bc^
EGCG 0.06%	167.85 ± 3.04 ^c^	192.00 ± 8.18 ^c^	0.13 ± 0.06 ^c^	4.14 ± 0.14 ^c^	3.30 ± 0.44 ^c^
EGCG 0.08%	95.81 ± 5.71 ^d^	134.67 ± 3.51 ^d^	0.08 ± 0.03 ^c^	3.47 ± 0.16 ^d^	1.73 ± 0.25 ^d^
EGCG 0.1%	98.61 ± 4.18 ^d^	104.67 ± 4.04 ^e^	0.04 ± 0.01 ^c^	3.50 ± 0.05 ^d^	1.43 ± 0.12 ^d^

The same indicator annotated with different letters indicates the significant difference (*p* < 0.05); the same letters indicate the difference is not significant (*p* > 0.05).

**Table 3:** Relaxation time T_2_ and corresponding peak area proportions of surimi gels with different amounts of EGCG.

Gels	T_2b_/ms	T_21_/ms	T_22_/ms	T_23_/ms	P_2b_	P_21_ + P_22_	P_23_
Control	0.647 ± 0.450 ^a^	3.569 ± 0.479 ^ab^	32.297 ± 0.849 ^a^	815.10 ± 5.405 ^c^	0.113 ± 0.023 ^d^	96.322 ± 0.072 ^c^	3.564 ± 0.083 ^c^
EGCG 0.02%	0.623 ± 0.093 ^a^	1.886 ± 0.032 ^b^	16.578 ± 2.118 ^c^	2130.0 ± 267.74 ^b^	0.556 ± 0.071 ^cd^	98.958 ± 0.057 ^a^	0.486 ± 0.119 ^d^
EGCG 0.04%	0.657 ± 0.484 ^a^	1.219 ± 0.148 ^b^	22.266 ± 0.935 ^b^	2863.8 ± 117.260 ^a^	1.093 ± 0.059 ^b^	97.781 ± 0.241 ^b^	1.126 ± 0.057 ^d^
EGCG 0.06%	1.037 ± 0.175 ^a^	5.821 ± 2.228 ^a^	23.961 ± 1.658 ^b^	564.85 ± 22.857 ^cd^	1.217 ± 0.258 ^b^	83.786 ± 0.499 ^d^	14.997 ± 0.564 ^b^
EGCG 0.08%	1.049 ± 0.468 ^a^	3.331 ± 0.815 ^ab^	24.129 ± 1.949 ^b^	431.13 ± 11.035 ^d^	0.726 ± 0.193 ^bc^	83.717 ± 0.470 ^d^	15.556 ± 0.372 ^b^
EGCG 0.1%	0.323 ± 0.227 ^a^	3.349 ± 0.914 ^ab^	23.866 ± 1.528 ^b^	394.59 ± 4.988 ^d^	2.309 ± 0.303 ^a^	78.396 ± 0.613 ^e^	19.295 ± 0.663 ^a^

The same indicator annotated with different letters indicates a significant difference (*p* < 0.05); the same letters indicate the difference is not significant (*p* > 0.05).

## Data Availability

The original contributions presented in the study are included in the article, further inquiries can be directed to the corresponding author.

## References

[1] Buda U., Priyadarshini M.B., Majumdar R.K., Mahanand S.S., Patel A.B., Mehta N.K. (2021). Quality characteristics of fortified silver carp surimi with soluble dietary fiber: Effect of apple pectin and konjac glucomannan. Int. J. Biol. Macromol..

[2] Li J.L., Munir S., Yu X.Y., Yin T., You J., Liu R., Xiong S.B., Yang H. (2020). Double-crosslinked effect of TGase and EGCG on myofibrillar proteins gel based on physicochemical properties and molecular docking. Food Chem..

[3] Alipour H.J., Rezaei M., Shabanpour B., Tabarsa M. (2018). Effects of sulfated polysaccharides from green alga Ulva intestinalis on physicochemical properties and microstructure of silver carp surimi. Food Hydrocoll..

[4] Takeuchi T. (2014). Progress on larval and juvenile nutrition to improve the quality and health of seawater fish: A review. Fish. Sci..

[5] Balange A., Benjakul S. (2009). Enhancement of gel strength of bigeye snapper (*Priacanthus tayenus*) surimi using oxidised phenolic compounds. Food Chem..

[6] Arsyad M.A., Akazawa T., Ogawa M. (2018). Effects of olive leaf powder on mechanical properties of heat-induced surimi gel. J. Aquat. Food Prod. Technol..

[7] Jongberg S., Terkelsen L.D.S., Miklos R., Lund M.N. (2015). Green tea extract impairs meat emulsion properties by disturbing protein disulfide cross-linking. Meat Sci..

[8] Jia N., Wang L.T., Shao J.H., Liu D.Y., Kong B.H. (2017). Changes in the structural and gel properties of pork myofibrillar protein induced by catechin modification. Meat Sci..

[9] Lai W.F., Baig M.M.F.A., Wong W.T., Bao T.Z. (2020). Epigallocatechin-3-gallate in functional food development: From concept to reality. Trends Food Sci. Technol..

[10] Tian Z.H., Jiang X., Xiao N.Y., Zhang Q., Shi W.Z., Guo Q.Y. (2022). Assessing the gel quality and storage properties of hypophythalmalmichthys molitrix surimi gel prepared with epigallocatechin gallate subject to multiple freeze-thaw cycles. Foods.

[11] Yuan Y., Zhao Y.Q., Yang X.Q., Li L.H., Wu Y.Y., Wei Y., Cen J.W. (2019). Cryoprotective mechanism of epigallocatechin gallate on frozen Nile tilapia (*Orechromis niloticus*) surimi. Food Sci..

[12] Utrera M., Estévez M. (2012). Analysis of tryptophan oxidation by fluorescence spectroscopy: Effect of metal-catalyzed oxidation and selected phenolic compounds. Food Chem..

[13] Liang F., Zhu Y.J., Ye T., Jiang S.T., Lin L., Lu J.F. (2020). Effect of ultrasound assisted treatment and microwave combined with water bath heating on gel properties of surimi-crabmeat mixed gels. LWT—Food Sci. Technol..

[14] Yan B.W., Jiao X.D., Zhu H.P., Wang Q., Huang J.L., Zhao J.X., Cao H.W., Zhou W.G., Zhang W.H., Ye W.J. (2020). Chemical interactions involved in microwave heat-induced surimi gel fortified with fish oil and its formation mechanism. Food Hydrocoll..

[15] Alakhrash F., Anyanwu U., Tahergorabi R. (2016). Physicochemical properties of Alaska pollock (*Theragra chalcograma*) surimi gels with oat bran. LWT—Food Sci. Technol..

[16] Tang S.W., Feng G.X., Gao R.C., Ren J.Y., Zhou X.D., Wang H.Y., Xu H., Zhao Y.H., Zeng M.Y. (2019). Thermal Gel Degradation (Modori) in Sturgeon (*Acipenseridae*) Surimi Gels. J. Food Qual..

[17] Jia N., Lin S.W., Wang L.T., Liu D.Y. (2020). Effects of changes in sulfhydryl content and surface hydrophobicity of myofibrillar protein induced by gallic acid on its gel properties. Food Sci..

[18] Cao Y.G., Xiong Y.L. (2015). Chlorogenic acid-mediated gel formation of oxidatively stressed myofibrillar protein. Food Chem..

[19] Sakamoto H., Kumazawa Y., Toiguchi S., Seguro K., Soeda K., Motoki M. (2006). Gel strength enhancement by addition of microbial transglutaminase during onshore surimi manufacture. J. Food Sci..

[20] Tang C.B., Zhang W.G., Zou Y.F., Xing L.J., Zheng H.B., Xu X.L., Zhou G.H. (2017). Influence of RosAprotein adducts formation on myofibrillar protein gelation properties under oxidative stress. Food Hydrocoll..

[21] Sano T., Noguchi S.F., Marsumoto J.J., Tsuchiya T. (1990). Thermal gelation characteristics of myosin subfragments. J. Food Sci..

[22] Salehi F., Kashaninejad M. (2014). Effect of different drying methods on rheological and textural properties of balangu seed gum. Dry. Technol..

[23] Gil B., Yoo B. (2014). Effect of salt addition on gelatinization and rheological properties of sweet potato starch-xanthan gum mixture. Starch Stärke.

[24] Liu R., Zhao S.M., Xiong S.B., Xie B.J., Liu H.M. (2007). Studies on fish and pork paste gelation by dynamic rheology and circular dichroism. J. Food Sci..

[25] Zhou X.X., Lin H.H., Zhu S.C., Xu X., Lyu F., Ding Y.T. (2020). Textural, rheological and chemical properties of surimi nutritionally-enhanced with lecithin. LWT—Food Sci. Technol..

[26] Sun F.Y., Huang Q.L., Hu T., Xiong S.B., Zhao S.M. (2014). Effects and mechanism of modified starches on the gel properties of myofibrillar protein from grass carp. Int. J. Biol. Macromol..

[27] Fan M.C., Huang Q.L., Zhong S.Y., Li X.X., Xiong S.B., Xie J., Yin T., Zhang B.J., Zhao S.M. (2019). Gel properties of myofibrillar protein as affected by gelatinization and retrogradation behaviors of modified starches with different crosslinking and acetylation degrees. Food Hydrocoll..

[28] Liu R., Zhao S.M., Xiong S.B., Xie B.J., Qin L.H. (2008). Role of secondary structures in the gelation of porcine myosin at different pH values. Meat Sci..

[29] Cao Y.G., True A.D., Chen J., Xiong Y.L. (2016). Dual role (anti-and pro-oxidant) of gallic acid in mediating myofibrillar protein gelation and gel in vitro digestion. J. Agric. Food Chem..

[30] Jiao X.D., Cao H.W., Fan D.M., Huang J.L., Zhao J.X., Yan B.W., Zhou W.G., Zhang W.H., Ye W.J., Zhang H. (2019). Effects of fish oil incorporation on the gelling properties of silver carp surimi gel subjected to microwave heating combined with conduction heating treatment. Food Hydrocoll..

[31] Shaarani S.M., Nott K.P., Hall L.D. (2006). Combination of NMR and MRI quantitation of moisture and structure changes for convection cooking of fresh chicken meat. Meat Sci..

[32] Hinrichs R., Götz J., Noll M., Wolfschoon A., Eibel H., Weisser H. (2004). Characterisation of different treated whey protein concentrates by means of low-resolution nuclear magnetic resonance. Int. Dairy J..

[33] Gravelle A.J., Marangoni A.G., Barbut S. (2016). Insight into the mechanism of myofibrillar protein gel stability: Influencing texture and microstructure using a model hydrophilic filler. Food Hydrocoll..

[34] Kudre T., Benjakul S., Kishimura H. (2013). Effects of protein isolates from black bean and mungbean on proteolysis and gel properties of surimi from sardine (*Sardinella albella*). LWT—Food Sci. Technol..

[35] Tokifuji A., Matsushima Y., Hachisuka K., Yoshioka K. (2013). Texture, sensory and swallowing characteristics of high-pressure-heat-treated pork meat gel as a dysphagia diet. Meat Sci..

[36] Jongberg S., Skov S.H., Tørngren M.A., Skibsted L.H., Lund M.N. (2011). Effect of white grape extract and modified atmosphere packaging on lipid and protein oxidation in chill stored beef patties. Food Chem..

[37] Li Y.C., Shi D.H., Zhang X.Y., Li X.H., Zou Q., Yi S.M. (2022). Gallic acid combined with ultrasound treatment improves the gel properties of *Lateolabrax japonicas* myofibrillar protein. Food Sci..

[38] Goormaghtigh E., Ruysschaert J.M., Raussens V. (2006). Evaluation of the information content in infrared spectra for protein secondary structure determination. Biophys. J..

[39] Tan M.T., Xie J. (2021). Exploring the effect of dehydration on water migrating property and protein changes of large yellow croaker (*Pseudosciaena crocea*) during frozen storage. Foods.

[40] Chanphai P., Bourassa P., Kanakis C.D., Tarantilis P.A., Polissiou M.G., Tajmir-Riahi H.A. (2018). Review on the loading efficacy of dietary tea polyphenols with milk proteins. Food Hydrocoll..

[41] Zhao Y.Y., Zhou G.H., Zhang W.G. (2019). Effects of regenerated cellulose fiber on the characteristics of myofibrillar protein gels. Carbohydr. Polym..

[42] Zhang L.L., Zhang F.X., Wang X. (2016). Changes of protein secondary structures of pollock surimi gels under high-temperature (100 °C and 120 °C) treatment. J. Food Eng..

[43] Nagy K., Courtet-Compondu M.-C., Williamson G., Rezzi S., Kussmann M., Rytz A. (2012). Non-covalent binding of proteins to polyphenols correlates with their amino acid sequence. Food Chem..

[44] Quan T.H., Benjakul S., Sae-leaw T., Balange A.K., Maqsood S. (2019). Protein-polyphenol conjugates: Antioxidant property, functionalities and their applications. Trends Food Sci. Technol..

